# Integrated gene profiling of fine‐needle aspiration sample improves lymph node metastasis risk stratification for thyroid cancer

**DOI:** 10.1002/cam4.5770

**Published:** 2023-03-14

**Authors:** Weituo Zhang, Xinwei Yun, Tianyu Xu, Xiaoqing Wang, Qiang Li, Tiantian Zhang, Li Xie, Suna Wang, Dapeng Li, Xi Wei, Yang Yu, Biyun Qian

**Affiliations:** ^1^ Hongqiao International Institute of Medicine, Shanghai Tong Ren Hospital and Clinical Research Institute, Shanghai Jiao Tong University School of Medicine Shanghai China; ^2^ National Clinical Research Center for Cancer, Key Laboratory of Cancer Prevention and Therapy, Tianjin's Clinical Research Center for Cancer Tianjin Medical University Cancer Institute and Hospital Tianjin People's Republic of China; ^3^ Shanghai Clinical Research Promotion and Development Center Shanghai Hospital Development Center Shanghai China

**Keywords:** biomarker, fine‐needle aspiration, machine learning, multi‐omics, risk stratification, thyroid cancer

## Abstract

**Background:**

Lymph node metastasis risk stratification is crucial for the surgical decision‐making of thyroid cancer. This study investigated whether the integrated gene profiling (combining expression, SNV, fusion) of Fine‐Needle Aspiration (FNA) samples can improve the prediction of lymph node metastasis in patients with papillary thyroid cancer.

**Methods:**

In this retrospective cohort study, patients with papillary thyroid cancer who went through thyroidectomy and central lymph node dissection were included. Multi‐omics data of FNA samples were assessed by an integrated array. To predict lymph node metastasis, we built models using gene expressions or mutations (SNV and fusion) only and an Integrated Risk Stratification (IRS) model combining genetic and clinical information. Blinded histopathology served as the reference standard. ROC curve and decision curve analysis was applied to evaluate the predictive models.

**Results:**

One hundred and thirty two patients with pathologically confirmed papillary thyroid cancer were included between 2016–2017. The IRS model demonstrated greater performance [AUC = 0.87 (0.80–0.94)] than either expression classifier [AUC = 0.67 (0.61–0.74)], mutation classifier [AUC = 0.61 (0.55–0.67)] or TIRADS score [AUC = 0.68 (0.62–0.74)] with statistical significance (*p* < 0.001), and the IRS model had similar predictive performance in large nodule [>1 cm, AUC = 0.88 (0.79–0.97)] and small nodule [≤1 cm, AUC = 0.84 (0.74–0.93)] subgroups. The genetic risk factor showed independent predictive value (OR = 10.3, 95% CI:1.1–105.3) of lymph node metastasis in addition to the preoperative clinical information, including TIRADS grade, age, and nodule size.

**Conclusion:**

The integrated gene profiling of FNA samples and the IRS model developed by the machine‐learning method significantly improve the risk stratification of thyroid cancer, thus helping make wise decisions and reducing unnecessary extensive surgeries.

## Graphical abstract

An Integrated Risk Score (IRS) model was developed to predict the risk of papillary thyroid cancer lymph node metastasis based on Fine‐Needle Aspiration samples' multi‐omics data. The IRS model achieved high AUC (0.87), sensitivity (0.86), and specificity (0.78), well calibrated, and improved clinical benefit in clinical decision analysis, thus helping make wise decisions preoperatively and reducing unnecessary extensive surgeries.

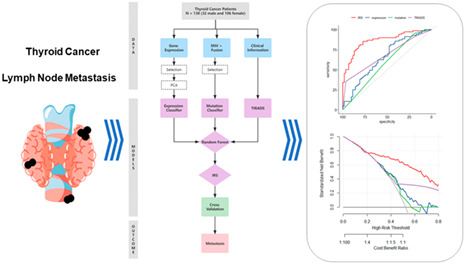

## 1 INTRODUCTION

According to recent cancer statistics, thyroid cancer is the most prevalent endocrine tumor, and its incidence is rapidly increasing.^1^ Over the past decade of data, the majority of the increase is attributable to papillary thyroid cancer (PTC), especially papillary thyroid microcarcinomas (PTMC).^2^ Previous findings suggest that although PTC/PTMC generally has a favorable prognosis, tumor metastasis and recurrence still occur in 3.6%–71.4% of patients with PTC/PTMC in different risk categories.^3^, ^4^ Identifying risk factors for lymph node metastases (LNM) assists surgeons in examining lymph node status and deciding if central lymph node dissection is required.^2^, ^3^, ^5^ Therefore, the risk stratification of thyroid cancer, such as the CUT score, is crucial for clinical decision‐making regarding surgical management, balancing the potential benefit and adverse outcomes of extensive surgery.^6^

Cytopathology of fine‐needle aspiration (FNA) samples is a standard method for thyroid cancer diagnosis.^7^ The Bethesda system suggests surgical lobectomy or thyroidectomy for FNA samples with Bethesda grades 4–6.^8^, ^9^ However, its ability to predict lymph node metastasis still needs improvement.^7^, ^10^, ^11^ Thus, the gene profiling of the FNA sample is considered a complementary method of cytopathology. The gene‐expression classifiers showed a significant diagnosis value among thyroid nodules with indeterminate cytology,^12^, ^13^, ^14^ achieving a sensitivity of 94% and specificity of 82% in prospective multi‐centered validation.^14^ Other researchers have assessed the diagnostic value of gene mutations.^15^, ^16^ It was also reported that a classifier combining the expression and mutation achieved sensitivity and specificity of 89% and 85%, respectively, for thyroid cancer diagnosis.^17^ As for the thyroid cancer risk stratification in the sense of invasion and lymph node metastasis, several genetic characteristics have been investigated.^18^, ^19^, ^20^, ^21^ Particularly, Tao et al^20^ reported the predictive value of BRAF V600E mutation for lymph node metastasis among PTC patients. However, the discrepancy between genotype and clinical outcome is very common. Utilizing multi‐omics data is a potential way to fill the gap, but till now, research targeting this area is scarce.^22^, ^23^

On the other hand, most of the current risk stratification models in medicine are developed using linear methods like logistic regression (LR) or linear discriminant analysis (LDA). When integrating large amounts and different types of information, these methods' limitations become apparent. Machine learning algorithms, such as random forest (RF) and artificial neural networks (ANN), can overcome their limitations and achieve superior prediction performance.^24^

This study was conducted to test the hypothesis that the integrated gene profiling of FNA samples and a predictive model constructed by machine learning method would improve the risk stratification of thyroid cancer, thereby guiding surgical decision‐making more effectively.

## 2 METHODS AND MATERIALS

### 2.1 Patients and specimens

We performed a retrospective cohort study involving 132 thyroid cancer patients at Tianjin Medical University Cancer Hospital, China, between 2016–2017, who met the following eligibility criteria: (1) 18 years or older; (2) underwent total thyroidectomy/lobectomy and central lymph node dissection (with/without lateral neck lymph node dissection); (3) had preoperative FNA samples with Bethesda grades 3 or 4; (4) pathologically confirmed thyroid cancer.

FNA of the thyroid was performed routinely under ultrasound guidance. The ultrasound images were evaluated by an independent, experienced sonographer according to the Thyroid Imaging Reporting and Data System (TI‐RADS) published by the Committee of the American College of Radiology (Version 2017). Cervical lymphadenectomy was typically performed for lymph nodes according to the Guidelines for diagnosis and treatment of thyroid nodules and differentiated thyroid cancer (2012, China). Cytopathology and histopathology were accessed by blinded experts. FNA samples and post‐surgery para‐nodule tissues of all patients were archived and accessed through gene profiling. Five patients were excluded because their samples did not meet the quality criteria.

This study was approved by the institutional review board (IRB) of Tianjin Medical University Cancer Hospital. All participants provided written informed consent.

### 2.2 Laboratory methods

Extraction of DNA/RNA was performed with the Mag‐bind blood and tissue DNA HDQ 96 kit and Mag‐bind Total RNA 96 kit (Omega Bioservices), respectively. Reverse Transcription by the RevertAid First Strand cDNA Synthesis Kit (Thermo Fisher Scientific). DNA/RNA purity was detected by a UV spectrophotometer (Nano Drop Technologies), and quantification was performed using the Qubit 3.0.

The library was prepared using the Uthyroid TM panel, which consists of DNA, RNA fusion, and RNA expression libraries. After adding barcodes and adaptors for NextSeq (Illumina) using PCR reagents, the quality of PCR products was checked with LabChip GX Touch24 (PerkinElmer). The sequencing process was carried out according to Illumina's protocols (Illumina) using NextSeqCN500 (BerryGenomics) 300 cycle reagent cartridge corresponding to 2 × 150 bp paired‐end configuration. The average depth of each run was over 500×.

### 2.3 Model development and evaluation

**FIGURE 1:**
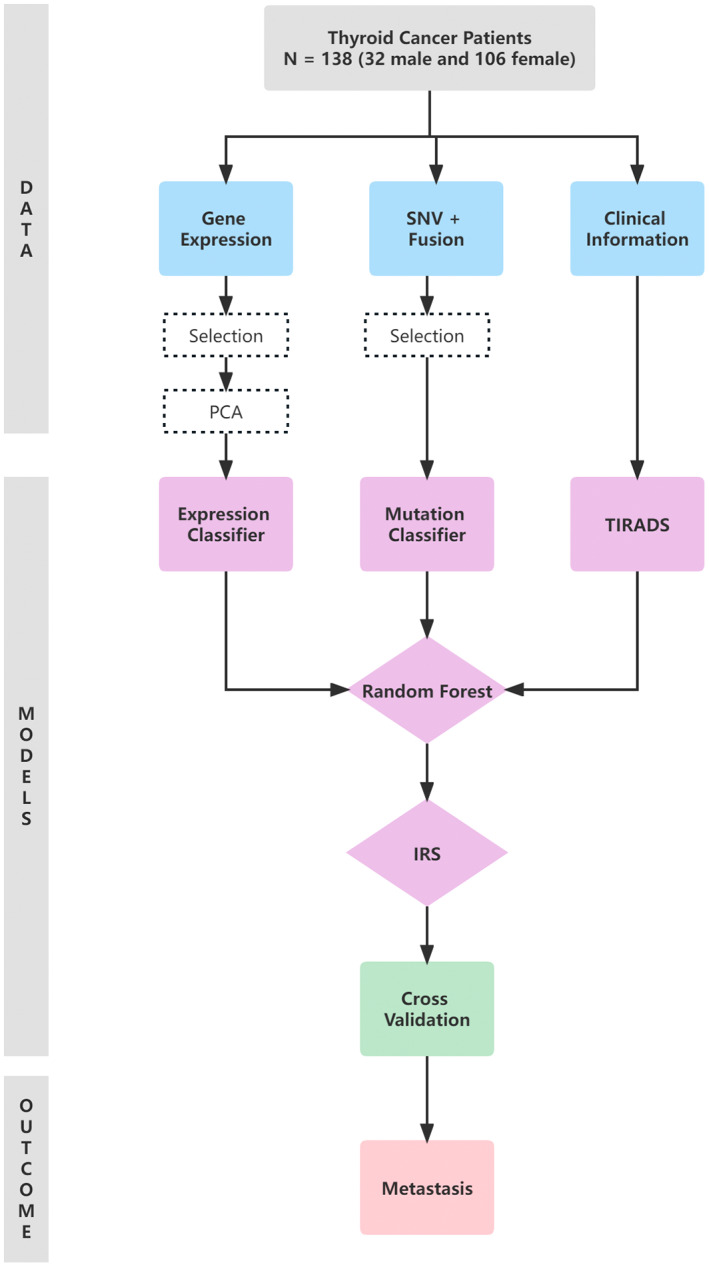
Flowchart of this study, demonstrating the data collection and machine‐learning model development process.

The overall pipeline of data collection, processing, and model development is described in Figure 1. In this study, tri‐fold data were collected: (1) Gene‐expression data from sequencing, (2) Gene SNV and fusion data from sequencing, (3) Demographic and preoperative clinical information of the patients, including age, sex, nodule size, TIRADS grade, etc. The omics data underwent the following preprocessing procedure. Adjusted *p*‐value (FDR) and fold‐change were used for preliminarily feature selection, as shown in volcano plots (Figure S1). Principle Component Analysis (PCA) was used for further dimension reduction. Principle components (PC) with eigenvalue >1 were retained (Figure S2).

We took two steps to integrate information and develop predictive models. First, the gene expression classifier and mutation classifier were built separately using corresponding genomic features. Second, the random forest (RF) algorithm served as a powerful integration method by which all types of genetic features and preoperative clinical information were combined to obtain the Integrated Risk Stratification (IRS) model.

In the end, we evaluated three predictive models we built: the expression classifier, mutation classifier, and the IRS model, and we also compared them with the TIRADS grade. For all the predictive models, k‐fold cross‐validation (*k* = 10) was used to evaluate their performance. The model development and evaluation were done with Python (version 3.6.2).

### 2.4 Statistical analysis

Categorical data were summarized with frequencies and percentages, and continuous data were summarized with mean (standard deviation) or medians (interquartile ranges) as appropriate. The area under the curve (AUC) of the receiver's operative curve (ROC), sensitivity, and specificity were calculated using established methods. AUCs in this study were compared using Delong's method. Clinical usefulness and net benefit were estimated by decision curve analysis (DCA).^25^ A multivariate logistic regression model was applied to test the significance of risk factors. A two‐tailed *p* < 0.05 was considered statistically significant. The derivation, validation, and reporting of prediction models followed the TRIPOD statement. Statistical analysis was done with R software, version 3.5.1 (The R Foundation for statistical computing).

## 3 RESULTS

### 3.1 Clinical characteristics of patients

Clinical characteristics (age at surgery, sex, nodule size, TIRADS, and lymph node metastasis) of the 132 included patients with thyroid cancer were presented in Table 1. Notably, nearly half of the nodules (59/132, 44.7%) were smaller than (or equal to) 1 cm. There were 68 patients (51.5%) who had lymph node metastasis, while 64 (48.5%) had not.

**TABLE 1:** Clinical characteristics of patients.

Characteristics	Value^a^ (*n* = 132)
Age (years)	44.8 (10.6)
Sex	
Male	31 (23.5%)
Female	101 (76.5%)
Nodule size (cm)	1.0 (0.7–1.5)
Nodule size group	
≤1.00 cm	59 (44.7%)
>.00 cm	73 (55.3%)
TIRADS	
IV A	18 (13.6%)
IV B	55 (41.7%)
IV C	33 (25.0%)
V	26 (19.7%)
Lymph nodes metastasis	
No	64 (48.5%)
Yes	68 (51.5%)

^a^ Data are shown as *n* (%), median (IQR), or mean (SD) as appropriate.

### 3.2 Mutation frequencies identified in samples

Table 2 demonstrates the gene mutation frequencies in FNA samples with and without lymph node metastasis. As shown in the table, among 132 FNA samples, genetic sequencing revealed 95 mutations in 90 samples. BRAF was the most common mutation (62.1% in FNA samples). Patients with lymph node metastasis had significantly higher frequency (50/68, 73.5%) than those without lymph node metastasis (29/65, 45.3%, OR = 3.5, *p* = 0.001). Other mutations were also more frequent in patients with lymph node metastasis, although without statistical significance.

**TABLE 2:** Mutation detected in FNA samples.

Mutation frequency^a^	FNA meta (*N* = 68)	FNA no‐meta (*N* = 64)	FNA all (*N* = 132)
BRAF	50 (73.5%)	29 (45.3%)	79 (62.1%)
NRAS	0 (0%)	0 (0%)	0 (0%)
TERT	1 (1.5%)	0 (0%)	1 (0.8%)
HRAS	1 (1.5%)	0 (0%)	1 (0.8%)
RET	2 (2.9%)	0 (0%)	2 (1.5%)
CCDC6 RET	3 (4.4%)	1 (1.6%)	4 (3.0%)
NCOA4 RET	2 (2.9%)	1 (1.6%)	3 (2.3%)
ETV6 NTRK3	2 (4.4%)	2 (3.1%)	4 (3.0%)
TPM3 NTRK1	1 (1.5%)	0 (0%)	1 (0.8%)

Abbreviation: FNA, fine‐needle aspiration.

^a^ All types of mutations detected in 132 samples are reported here, which reveals the difference in mutation frequency in different sample sets. The columns represented (from left to right) FNA samples from patients with lymph node metastasis, FNA samples from patients without lymph node metastasis, and all FNA samples, respectively.

### 3.3 Model performance evaluation

**FIGURE 2:**
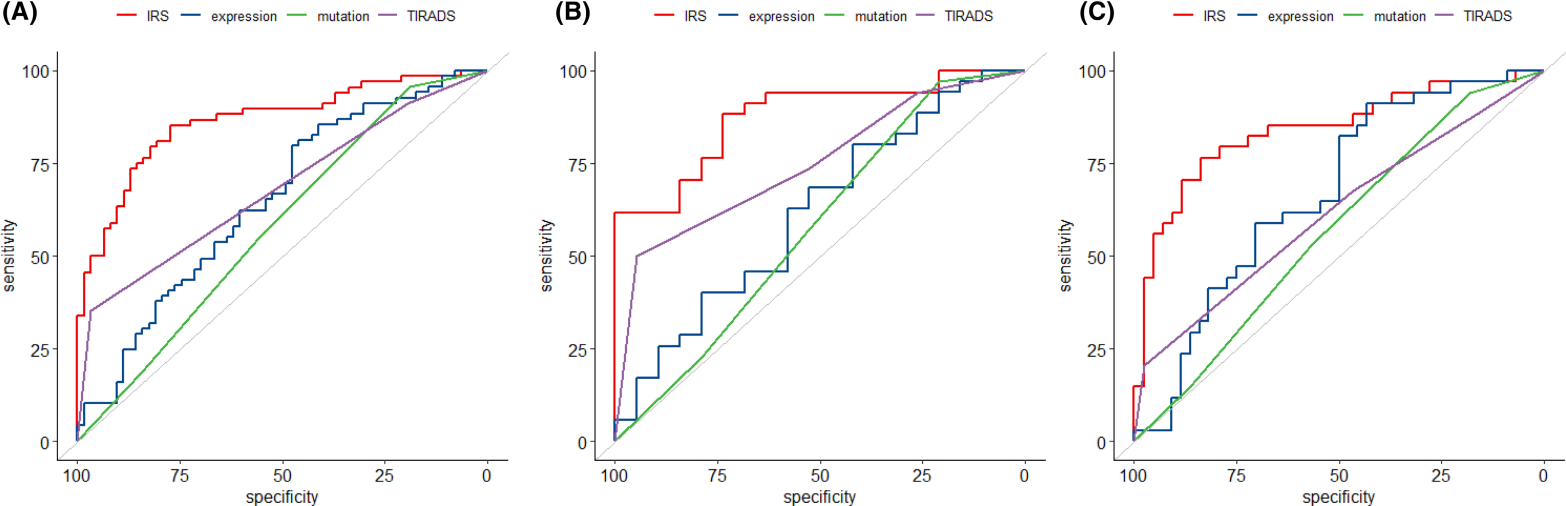
The receiver operating characteristic (ROC) curves of the models predicting lymph node metastasis in all patients (panel A), nodules >1 cm (panel B), and nodules ≤1 cm (panel C). The Red lines stand for the integrated risk stratification (IRS) model; the blue lines for the expression classifier; the green lines for the mutation classifier; the purple line for the TIRADS score; the gray lines for reference.

Figure 2A compares the ROC curves of predictive models we built (expression classifier, mutation classifier, and IRS model) and the TIRADS score as a baseline reference. The AUC of ROCs, the sensitivities, and the specificities were summarized in Table 3. Testing the AUCs via Delong's method, all models demonstrated significant predictive value compared with random guess reference (*p* < 0.001). The IRS model demonstrated greater performance (AUC = 0.87, 95% CI = 0.80–0.94) than either expression classifier (AUC = 0.67, 95% CI = 0.61–0.74), mutation classifier (AUC = 0.61, 95% CI = 0.55–0.67) and TIRADS score (AUC = 0.68, 95% CI = 0.62–0.74) with statistical significance (*p* < 0.001). The optimal diagnostic cut‐off of the IRS model was 0.5, yielding a sensitivity of 0.86 (CI: 0.78–0.89) and a specificity of 0.78 (CI: 0.72–0.84). The two genetic classifiers (expression, mutation) had higher sensitivities but lower specificities compared with the TIRADS score, thus potentially having complementary predictive value. Figure 2B,C show the model ROCs in subpopulations of large nodule size (>1 cm) and small nodule size (≤1 cm). The IRS model had similar predictive performance in large nodule (AUC = 0.88, 95% CI = 0.79–0.97) and small nodule (AUC = 0.84, 95% CI = 0.74–0.93) subgroups. Detailed AUCs of models in subgroups are given in Table S2. The calibration plot (Figure S3) shows that all predictive models (IRS, expression classifier, and mutation classifier) were well calibrated, that is, the model predicted lymph node metastasis risk is consistent with the empirically observed risk.

**TABLE 3:** Summary of model performance.

Models	AUC (95% CI)	Sensitivity (95% CI)	Specificity (95% CI)
IRS model	0.87 (0.80–0.94)^a^	0.86 (0.78–0.89)	0.78 (0.72–0.84)
expression classifier	0.67 (0.61–0.74)^a^	0.802 (0.72–0.87)	0.48 (0.42–0.54)
mutation classifier	0.61 (0.55–0.67)^a^	0.96 (0.91–0.98)	0.21 (0.17–0.26)
TIRADS	0.68 (0.62–0.74)^a^	0.36 (0.31–0.42)	0.87 (0.83–0.91)

Abbreviations: AUC, area under curve; CI, confidence interval; IRS, integrated risk stratification.

^a^ The AUC is significantly >0.5 (reference: random guess), *p* < 0.05.

### 3.4 Gene profiling has independent prognostic value in addition to clinical information

To further access the predictive value of gene profile and clinical information, we performed a multivariate logistic regression of the lymph node metastasis risk. As the expression and mutation classifier were strongly correlated, the linear model failed to include both of them as risk factors. Therefore, the genetic risk, a combination of these two classifiers, was used in the regression. The clinical characteristics, including age, sex, nodule size, TIRADS grade, and unifocal/multifocal, were considered potential risk factors. Table 4 shows the risk factors demonstrated significant association with lymph node metastasis in multivariate analysis. Genetic risk and TIRADS = 5 were the predominant risk factors. The genetic risk had an odds ratio of 10.29 (CI: 1.13–105.31), and TIRADS = 5 had an odds ratio of 41.12 (5.89–857.36), respectively. Big nodule size (>1 cm), TIRADS 4b, and 4c (taking TIRADS 4a as standard reference) were risk factors with moderate effect, while age was a protective factor. These findings are consistent with previous literature. We conclude the genetic risk from multi‐omics data of FNA samples had additional prognostic value for thyroid cancer in the presence of other preoperative clinical information.

**TABLE 4:** Multivariate analysis for risk factors of lymph node metastasis.

Factors	*β*	OR	95% CI	*p*‐value
Genetic risk	2.33	10.29	1.13–105.31	0.042*
TIRADS				
4a	Reference	–	–	–
4b	1.61	3.20	0.94–12.22	0.073
4c	0.77	2.16	0.61–8.38	0.246
5	3.72	41.12	5.89–857.36	<0.001***
Age	−0.05	0.96	0.96–1.00	0.039*
Nodule size group				
≤1 cm	Reference	–	–	–
>1 cm	0.85	2.33	1.03–5.44	0.046*

*Note*: The genetic risk, TIRADS grade, age, and nodule size are shown to be independent risk factors of lymph node metastasis. The genetic risk is a combination of expression and mutation classifiers.

Abbreviations: CI, confidence interval; OR, odds ratio; TIRADS,thyroid imaging reporting & data system.

* Significance *p* < 0.05

*** Significance *p* < 0.001.

Furthermore, we listed the top 10 genetic features which contributed the most to the IRS prediction model regarding the relative importance score from the random forest (Table S1) and the top 10 differentiated genetic features between the paired malignancy versus para‐nodule samples. A discrepancy was observed between the two top 10 genetic features listed.

### 3.5 Impact on clinical decision

**FIGURE 3:**
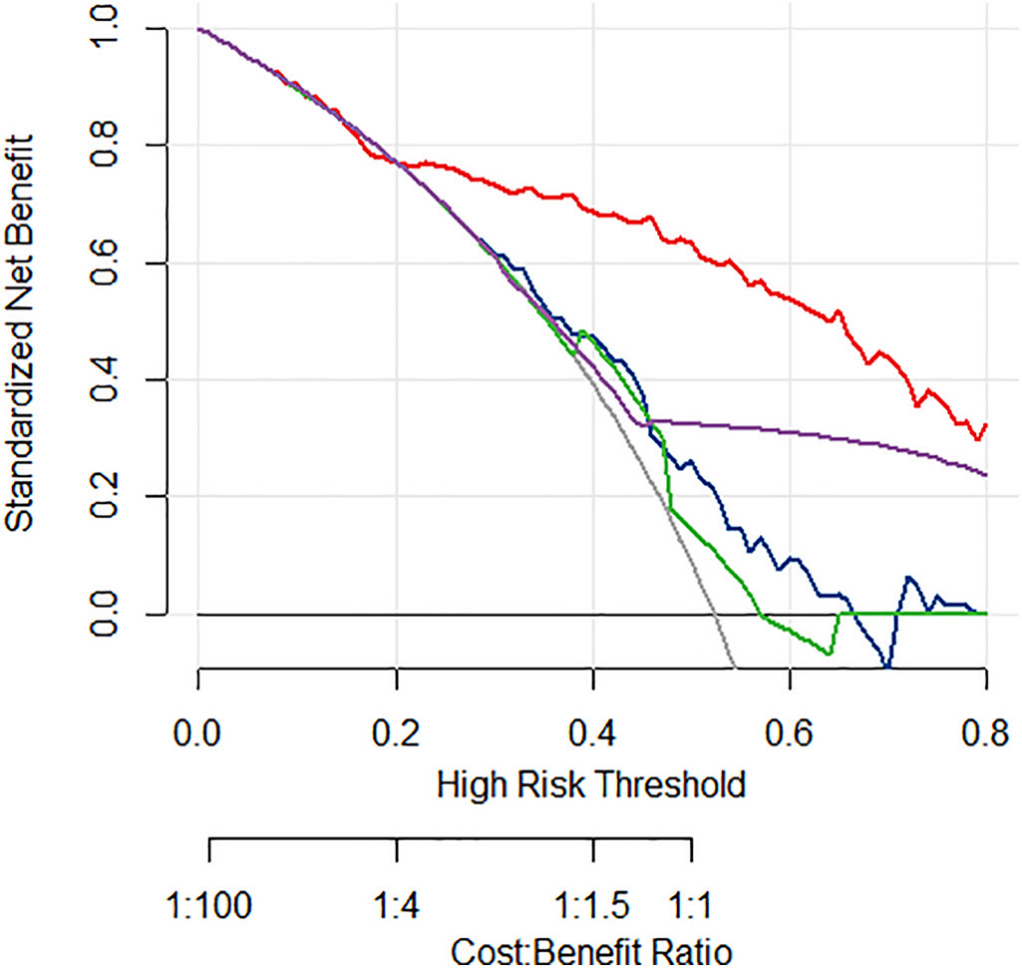
Decision analysis curve of predictive models, showing the net clinical benefit of lymph node dissection decision based on each predictive model. The Red lines stand for the integrated risk stratification (IRS) model; the blue lines for the expression classifier; the green lines for the mutation classifier; the purple line for the TIRADS score; gray line for the reference scenario that all patients have lymph node dissection.

To further assess the clinical impact on disease management, we applied decision curve analysis to our predictive model, as shown in Figure 3. Consider the two baseline clinical strategies “All” and “None.” “All” means all patients have extensive surgery (e.g., cervical lymphadenectomy), while “None” means no patient has extensive surgery. We also considered the model‐based strategies: each predictive model provides a family of strategies relying on a risk threshold p_c_, by which extensive surgery is only performed for patients with risk higher than p_c_. Each p_c_ also corresponds to a cost/benefit ratio (e.g., the harm of an unnecessary cervical lymphadenectomy/the benefit of a correct removal of the metastasis lymph node).

Figure 3 shows, compared with the “All” or “None” strategies, the risk stratifications using expression classifier, mutation classifier, or TIRADS separately only significantly increase net clinical benefit when *p*_c_ > 0.4 (i.e., cost/benefit ratio >1:1.5).

## 4 DISCUSSION

In the current clinical practice, the management strategy of papillary thyroid cancer varies in counties and hospitals. For instance, prophylactic central lymphadenectomy, for instance, remains controversial because it may reduce the likelihood of persistence/recurrence, but introduces additional risks of surgical complications.^26^, ^27^, ^28^ Our IRS model based on multi‐omics data of preoperative FNA samples provides a promising approach to making better clinical decisions for prophylactic central lymphadenectomy.

To the best of our knowledge, our study is the first to integrate FNA gene profiling (including expression, SNV, and fusion) for lymph node metastasis risk stratification in papillary thyroid cancer. We conceptually demonstrated that multi‐omics prediction is superior to expression or mutation prediction. In this study, the expression, SNV, and fusion of FNA samples were obtained through sequencing on one integrated platform (Uthyroid TM panel), which would make the utilization of multi‐omics data in clinical practice more applicable.

The associations between genetic profile and lymph node metastasis have already been reported,^18^, ^19^, ^20^, ^21^, ^22^, ^23^ but they have not yet been widely accepted to make personalized surgical decisions in actual clinical practice. Our decision curve analysis (DCA) partially explains why – the accuracy of these prediction models does not lead to clinical benefit in the realistic region of the cost/benefit ratio. Assessing the cost/benefit ratio of extensive surgery like cervical lymphadenectomy requires a cautious analysis of the risk of surgical complications, tumor recurrence, and corresponding consequences, etc., but this ratio is usually <1:1 in most scenarios of PTC. At this region of the cost–benefit curve, the expression classifier and mutation classifier have independent predictive values, but the predictions are insufficient to translate into clinical benefit.^25^ In contrast, the IRS model for lymph node metastasis not only has good predictive accuracy but also significantly improves the clinical benefit when *p* > 0.2 (i.e., cost/benefit >1:4). Thus, the IRS model is more likely to have a real impact on clinical decisions.

Our study population contained a fair portion of small‐size thyroid cancer (PTMC), for which gene profiling of FNA samples is scantly studied. There is a doubt that the FNA for PTMC is remarkably imprecise.^29^ In our study, the mutation frequency detected in our FNA samples was lower than those reported in the literature.^15^, ^16^ It may partly be due to the large proportion of PTMC. However, in the subgroup analysis, the IRS model, expression classifier, and mutation classifier have similar predictive performance in small nodules (≤1 cm) compared with large nodules (>1 cm). These findings indicate the feasibility and utility of FNA samples in PTMC. Furthermore, we observed a discrepancy between the most important genes for predicting lymph node metastasis in the IRS model and the most differentiated genes between the PTCs and normal tissues. This discrepancy enhanced our insight that the occurrence and metastasis of thyroid cancer might be driven by different sets of genes.

There are several limitations of our study. First, the sample size is limited, so we can only include a limited number of risk factors in our model to avoid over‐fitting, and our model was not externally validated. Second, our sample did not cover enough pathological diversity, all of our cases are PTC or PTMC. However, in the real clinical setting, histopathology is unavailable preoperatively. Thus the impact of patients with other pathological types should be considered when applying our model. Further studies with larger sample sizes and a more general population are required.

## 5 CONCLUSION

In this study, we developed an Integrated Risk Score (IRS) model predicting the risk of lymph node metastasis of papillary thyroid cancer based on multi‐omics data of FNA samples. The IRS model achieved high AUC (0.87), sensitivity (0.86), and specificity (0.78), well‐calibrated, and significantly improved clinical benefit in clinical decisions like prophylactic central lymphadenectomy. We conceptually proved the utility of integrating multi‐omics data and applying machine learning algorithms in this important clinical setting. Further study is required to validate this model in a larger and more diverse population.

## AUTHOR CONTRIBUTIONS

**Weituo Zhang:** Conceptualization (equal); data curation (equal); formal analysis (equal); funding acquisition (equal); writing – original draft (equal); writing – review and editing (equal). **Xinwei Yun:** Conceptualization (equal); data curation (equal); formal analysis (equal); resources (equal); writing – review and editing (equal). **Tianyu Xu:** Formal analysis (equal); software (equal); writing – review and editing (equal). **Xiaoqing Wang:** Data curation (equal); writing – review and editing (equal). **Qiang Li:** Formal analysis (equal). **Tiantian Zhang:** Formal analysis (equal). **Li Xie:** Writing – review and editing (equal). **Suna Wang:** Software (equal). **dapeng Li:** Data curation (equal). **Xi Wei:** Conceptualization (equal); data curation (equal); resources (equal); writing – review and editing (equal). **Yang Yu:** Conceptualization (equal); resources (equal); writing – review and editing (equal). **Biyun Qian:** Conceptualization (equal); data curation (equal); funding acquisition (equal); methodology (equal); project administration (equal); resources (equal); writing – review and editing (equal).

## CODE AVAILABILITY

The code supporting this study's findings is available from the corresponding authors upon reasonable request.

## CONFLICT OF INTEREST STATEMENT

The authors declare no competing interests.

## Supporting information


Appendix S1.

## Data Availability

All datasets used and analyzed are available by reasonable request from the corresponding author in this study.
