# Supplementary material for: Integrated gene profiling of fine‐needle aspiration sample improves lymph node metastasis risk stratification for thyroid cancer
Source: Cancer Med. 2023 Mar 14;12(9):10385–92. doi: 10.1002/cam4.5770 (PMC10225186; doi:10.1002/cam4.5770)
Supplement: Supplementary file 1 — Appendix S1. [file CAM4-12-10385-s001.docx]

**Supplementary Material**

**Figure S1：**Volcano plot for biomarker selection according to expression data.

The gene selection threshold is set to |log_2_(FC)| > 1 and p<0.00032 according to the Bonferroni correction. FC=fold change.

**A**


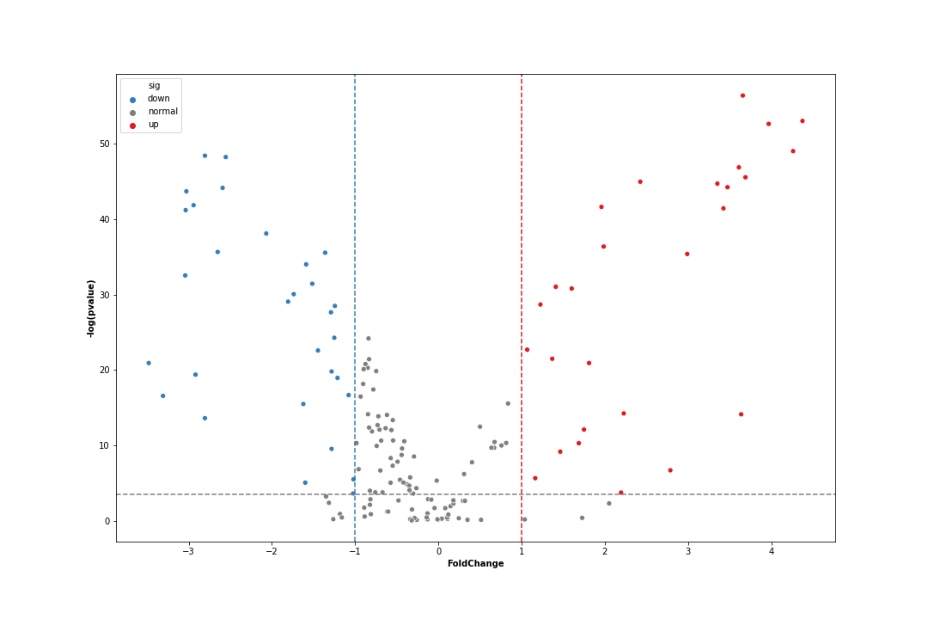


**B**

**
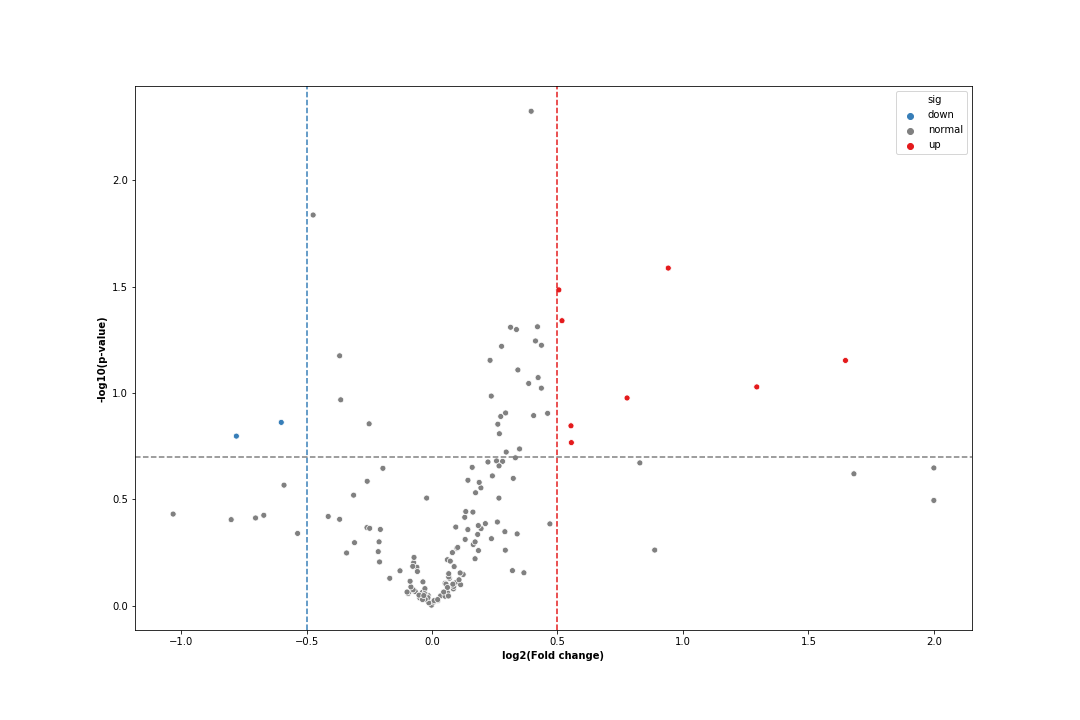
**

**Figure S2:** Score plot for the PC1 and PC2 of samples from PCA analysis. Each point in this plot represents a sample in the PC1 (x-axis) and PC2 (y-axis) coordinate.
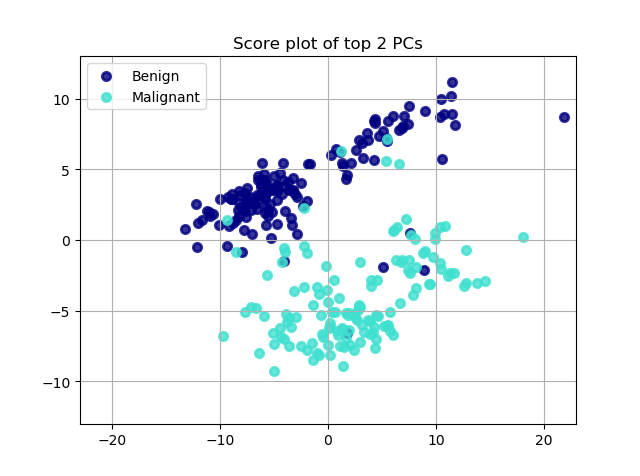


**Figure S3:** Calibration plot of models predicting lymph node metastasis. The model predicted risk vs. the empirically observed risk in our data. The Red lines stand for the integrated risk stratification (IRS) model; the blue lines for the expression classifier; the green lines for the mutation classifier; the grey lines for reference.


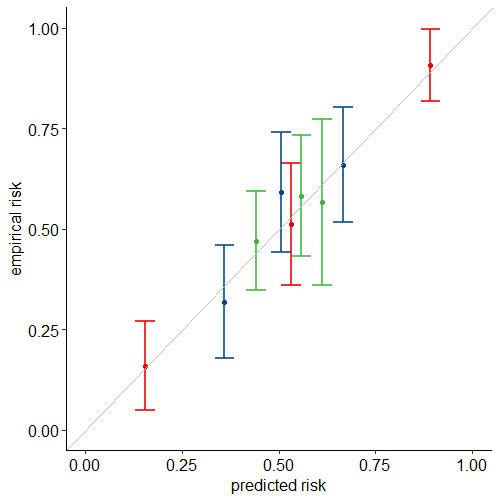


**Table S1:** The top 10 genetic features for predicting thyroid nodule malignancy and lymph node metastasis separately listed according to the importance score from Random Forest algorithm.

| **Predicting malignancy** | | **Predicting lymph node metastasis** | |
| --- | --- | --- | --- |
| **Gene** | **Relative importance** | **Gene** | **Relative importance** |
| **BRAF** | 1.00 | MT1G | 1.00 |
| SLC34A2 | 0.81 | **BRAF** | 0.79 |
| FN1 | 0.80 | MDK | 0.69 |
| PROS1 | 0.75 | PPP2R2B | 0.66 |
| DPP4 | 0.72 | TIMP1 | 0.59 |
| FABP4 | 0.71 | SLC24A5 | 0.57 |
| LIPH | 0.71 | NDUFC2 | 0.46 |
| MT1F | 0.71 | TENM1 | 0.33 |
| CD36 | 0.69 | SERGEF | 0.24 |
| CLDN16 | 0.69 | CFHR1 | 0.18 |

**Table S2：**The AUCs of ROC curves of the models predicting lymph node metastasis in all patients, nodules >1cm and nodules ≤1cm.

| **Models** | **All patients** | **nodule size >1cm** | **nodule size ≤1cm** |
| --- | --- | --- | --- |
| IRS model | 0.87 (0.80, 0.94) | 0.88 (0.79, 0.97) | 0.84 (0.74, 0.93) |
| expression classifier | 0.67 (0.61, 0.74) | 0.63 (0.47, 0.79) | 0.67 (0.55, 0.79) |
| mutation classifier | 0.61 (0.55, 0.67) | 0.60 (0.43, 0.76) | 0.59 (0.47, 0.71) |
| TIRADS | 0.68 (0.62, 0.74) | 0.75 (0.62, 0.88) | 0.62 (0.50, 0.73) |
